# Use of NIRS to explore skeletal muscle oxygenation during different training sessions in professional boxing

**DOI:** 10.1007/s00421-023-05305-1

**Published:** 2023-09-01

**Authors:** Andrew Usher, John Babraj

**Affiliations:** Department of Sport and Exercise Science, Abertay University, Bell St, Dundee, DD1 1HG Scotland

**Keywords:** NIRS, Rectus femoris, Oxygenation, Desaturation, Recovery

## Abstract

**Purpose:**

The physiological examination of boxing has been limited to systemic response in amateur athletes. The demands of professional boxing have been overlooked, despite the different competition format. We sought to determine the physiological demands placed on skeletal muscle in professional boxing.

**Methods:**

Ten male professional boxers (age 26 ± 5 years, height 177 ± 4 cm, weight 71 ± 6 kg) were recruited for this observational study. On different days, the athletes completed 6 × 3 min rounds of pad, bag or spar-based training with 1 min recovery between each round. Prior to each session, participants put on a heart rate monitor and near-infrared spectroscopy attached to the belly of the rectus femoris muscle to record heart rate and muscle oxygenation.

**Results:**

There were significantly less punches thrown in sparring compared to other training modalities (*p* < 0.001). Skeletal muscle oxygenation across training modalities consisted of a delay, fast desaturation and steady state. Across rounds there was a significant increase in time delay for desaturation (*p* = 0.016), rate of fast desaturation (*p* < 0.001) and duration of fast desaturation (*p* = 0.019). There was a significant difference in sparring for the heart rate where skeletal muscle oxygenation changes occurred compared to pads or bag sessions (*p* < 0.001).

**Conclusion:**

The findings highlight differences in the skeletal muscle response to the different training modalities. Practitioners need to be aware of the muscular demands of each session to allow optimal adaptation across a training camp. Training needs to allow the skeletal muscle to achieve a new oxygenation steady state rapidly to promote efficient performance across rounds.

## Introduction

Boxing athletes compete over varying weight classifications and round formats (amateur boxing 3 rounds, professional boxing, 4–12 rounds) with a typical duration of 3 min per round. Rounds have been characterised as high-intensity based with moments of active and passive recovery (Davis et al. 2015), demanding attributes such as multidirectional footwork, evasion, striking, tactical awareness and recovery between rounds (El Ashker 2011). The physiological demands of boxing have been carried out solely in amateur boxers with research in professional boxers limited to weight cutting or injury risk. This means the demands on a professional boxer remain unknown.

Amateur boxing research has shown that the work to rest ratio in rounds is higher in elite (18:1) than novice (9:1) boxers, with work to rest ratio increasing across rounds (Slimani et al. 2017). Research has primarily focussed on systemic physiology utilising conventional laboratory testing (graded exercise test), simulated boxing (bag and pad work) or post-sparring data (Finlay et al. 2020, 2022). During a graded exercise test, peak heart rate ranged from 178 to 204 bpm (El-Ashker and Nasr 2012; Ghosh and Goswami 1995), VO_2_ peak ranged from 41 to 65 ml kg^−1^ min^−1^ (Kravitz et al. 2003; Nassib et al. 2017) with peak blood lactate between 7 and 13 mmol l^−1^ (El-Ashker and Nasr 2012; Lal Khanna and Manna 2006) in amateur athletes. Striking a heavy bag or pads is a common training modality in boxing and has been utilised to create simulated boxing activity in research. Following simulated boxing rounds, peak heart rate ranged from 158 to 195 bpm, VO_2_ peak ranged from 42 to 55 ml kg^−1^ min^−1^ and blood lactate was between 2 and 17 mmol l^−1^ (De Lira et al. 2013; Finlay et al. 2020) in amateur boxers with all components increasing across subsequent rounds. Whilst the changes across rounds highlight increasing physiological demand, simulations do not fully represent technical bag or pad sessions due to their set choreography, nor do they take into consideration the ad hoc nature of competition or sparring. To date, there has been no research on the response of skeletal muscle during boxing training or competition in either amateur or professional boxing.

With the advancement of technology, Bluetooth short-range wireless near-infrared spectroscopy (NIRS) monitors allow determination of skeletal muscle demand in less controlled environments. NIRS, is a validated (A. Feldmann et al. 2019; Ryan et al. 2013) and non-invasive way to ascertain changes in muscle oxygen saturation (SmO_2_) and haemoglobin (Hb) within the muscle tissue, allowing real-time measurement to determine athletic performance and to monitor oxidative muscle metabolism (Marcon et al. 2011; Miura et al. 2003). In sport climbing, there is a rapid muscle desaturation with the onset of isometric hold followed by rapid recovery after the removal of the contraction (Dindorf et al. 2023) whereas continuous exercise produces a rapid muscle desaturation followed by a prolonged equilibrium at a lower muscle oxygen level (Hesford et al. 2013). During incremental cycle exercise, SmO_2_ has been shown to drop with increasing workload and correlated to lactate and ventilatory thresholds (A. Feldmann et al. 2022). In kayak athletes, SmO_2_ during a time trial has been shown to be a better predictor of performance than VO_2_ (Paquette et al. 2018)*,* demonstrating the need for greater understanding of peripheral demand over systemic delivery. To date, there is no data on the peripheral demands of boxing-based exercise. The aim of this observational study is to utilise NIRS to determine, for the first time, the physiological demand on the rectus femoris muscle in different training sessions (bag work, pad work and competitive sparring).

## Methods

### Subjects

Participants: ten male professional boxers (age 26 ± 5 years, height 177 ± 4 cm, mass: 71 ± 6 kg, body fat 10 ± 5%, rectus femoris fat thickness: 5.2 ± 2.0 mm) were recruited for this study. Weight classifications ranged from super lightweight to super welterweight with an average of 8 ± 4 professional bouts (range 2–15 bouts; median 7 bouts) and each athlete routinely used 10 × 3 min rounds in training sessions and 6 × 3 min rounds in sparring sessions. Each athlete held a current professional licence with the British Boxing Board of Control. Participants were excluded if there was a loss of training days over the last 3 months due to musculoskeletal injury or sanction from British Boxing Board of Control preventing the athlete from taking part in sparring. Participants were informed verbally and in writing on the risks and benefits of the study prior to signing informed consent form and was approved by Abertay University Research Ethics Committee (EMS4768). The study was carried out in line with the Declaration of Helsinki, except for the registration in a database.

### Design

This is an observational study of the physiological response to different training modalities in professional boxers. Data were collected on skeletal muscle oxygenation and heart rate on all participants across three different training modalities with the order of training the same for all participants: 1. heavy bag session, 2. technical pad session, 3. sparring session. There was a minimum of 24 h between each session. Training sessions were kept in the same order as the athletes would normally perform the heavy bag or pad sessions so that their training schedules were not altered. Sparring was always completed last as athletes needed to be paired by a boxing coach and had to be available at the same time.

### Body composition

All participants had height determined by stadiometer (Seca 264, Seca, UK) and body composition was determined by bioimpedance (Tanita MC-780, Tanita, Japan). The fat thickness on the rectus femoris muscle was determined by ultrasound (Bodymetrix, Intelametrix, USA) as a fat thickness of greater than 14 mm will impede NIRS signal (A. Feldmann et al. 2019).

### Training sessions

Prior to training sessions, athletes were advised to fast for 2 h, with no food or water consumed. Athletes were asked to perform their normal training routine in the days prior to the testing sessions. Upon arrival at the gym, the athletes had near-infrared spectroscopy (NIRS; Moxy Monitor, Fortiori Design LLC, USA) taped to the rectus femoris muscle of the left and right leg. The Moxy is a lightweight (48 g) and small (62 × 52 × 15 mm) device that uses continuous four wavelengths (680 nm, 720 nm, 760 nm, and 800 nm) reporting values of SmO_2_ in the form of a 0–100 percentage scale (A. M. Feldmann et al. 2020). This percentage is calculated via the equation:

$${\text{SmO}}2 = \frac{{{\text{Hb}} + {\text{O}}_{2} {\text{Mb}}}}{{\left( {{\text{O}}_{2} {\text{Hb}} + {\text{O}}_{2} {\text{Mb}}} \right) + \left( {{\text{HHb}} + {\text{HMb}}} \right) }}$$

The Moxy monitor was set to high speed, 0.5 s update with no smoothing. The Moxy monitor has been shown to be valid and reliable during exercise with a greater dynamic scale than the portamon monitor (Feldmann et al. 2019; McManus et al. 2018), and has been used to explore muscle oxygenation during a wide range of sporting environment and training domains (Paquette et al. 2021; Perrey and Ferrari 2018). Arterial occlusion of the femoral artery was carried out on five participants to determine responsiveness of the Moxy monitor SmO_2_ values. An inflation cuff was placed at the top of the leg and was rapidly inflated to > 300 mmHg. The time from cuff inflation to decline in rectus femoris muscle oxygenation was 0.319 ± 0.114 s.

Participants then attached a heart rate monitor (Polar H10, Polar Electro, UK). All data were then collected via Bluetooth transmission to the VO_2_ master app (VO2 Master Health Sensors Inc, Canada). Prior to each session, the athletes were instructed to carry out three rounds of 3-min shadow boxing with 1 min recovery, simulating boxing movements and striking. Each session was filmed to allow punch count to be determined.

1. Heavy bag session: Participants performed 6 × 3 min rounds with 1 min recovery between rounds, with athlete remaining standing during recovery. Participants were informed to carry out the heavy bag session in the same way as they would normally do a heavy bag session. No requirements were placed on the athlete around the number or type of punches to be thrown during each round.
2. Technical pad session: Participants performed 6 × 3 min rounds with 1 min recovery between rounds, with athlete remaining standing during recovery. Pad sessions were taking by the same British Boxing Board of Control professional licenced boxing coach to ensure consistency of pad work. The same pad drill was used with each athlete.
3. Sparring session: The participants were paired for competitive sparring based on their weight classification and perceived skill level by a British Boxing Board of Control professional licenced boxing coach to ensure consistency of spar across participants. Participants performed 6 × 3 min rounds with 1 min recovery between rounds, with athlete remaining standing during recovery.

### Data analysis

**Fig. 1:**
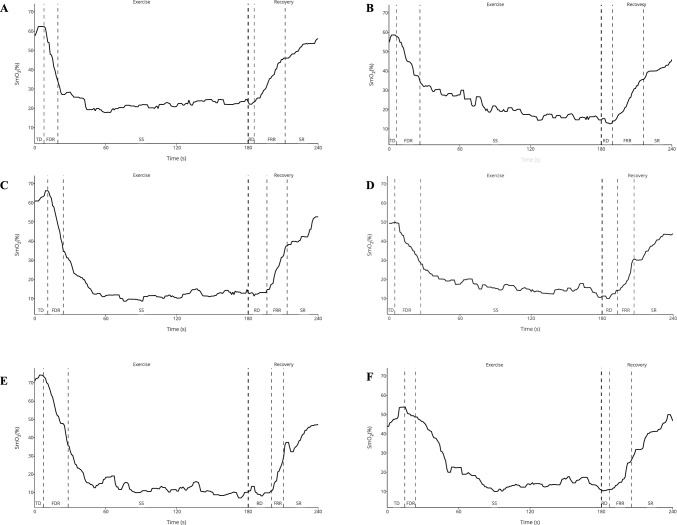
Example of desaturation and recovery curve. **A** Round 1 bag; **B** round 6 bag; **C** round 1 pad; **D** round 6 pad; **E** round 1 spar; **F** round 6 spar. *TD* time delay, *FRD* fast desaturation rate, *SS* steady state RD resaturation delay, *FRR* fast resaturation rate, *SR* slow recovery

Heart rate and SmO_2_ were exported from VO_2_ master as a 1 s average and processed in Python Jupyter Lab (version 3.3.2). A median average 5 s filter was applied to the data to smooth any movement artefacts (Buchheit and Ufland 2011). SmO_2_ was used for analysis as it gives a better indication of skeletal muscle oxygenation when blood flow is not steady (Buchheit and Ufland 2011). SmO_2_ data during rounds and recovery were plotted against time and heart rate and linear equations fitted to the components of the SmO_2_ curve. The time or heart rate at different points in the SmO_2_ curve were determined through solving for where the linear components meet (Fig. 1). During rounds, there were three clear components in the NIRS signal, initial time delay where oxygenation is stable, a fast desaturation where oxygenation levels are falling and a new equilibrium where oxygenation is lower but relatively consistent (Fig. 1). Variation in the heart rate to SmO_2_ linear curves was calculated using median absolute deviation as data are skewed in the steady state during exercise. Video footage was analysed by a Boxing Scotland level 2 boxing coach who manually counted punches for each training session. Total number of punches were counted for the initial time delay in muscle desaturation, during the fast desaturation and across the entire round (Fig. 1, Table 1).

**Table 1:** Punches thrown in different phases of the skeletal muscle response

	Bag punch count			Pad punch count			Spar punch count		
	TD	FDR	RT	TD	FDR	RT	TD	FDR	RT
Round 1	16 ± 13	24 ± 8^ g^	187 ± 56	7 ± 3	22 ± 11	172 ± 17^d,e^	3 ± 2^a,d^	9 ± 5^a^	58 ± 20^a^
Round 2	17 ± 13	29 ± 11	165 ± 32	9 ± 4	17 ± 8^c^	159 ± 28	4 ± 2^a^	6 ± 4^a,e^	60 ± 19^a^
Round 3	21 ± 18	34 ± 10	177 ± 43	10 ± 6	19 ± 10^c^	182 ± 55^d,e,f^	7 ± 5^b,f^	11 ± 10^a^	58 ± 17^a^
Round 4	20 ± 15^f^	31 ± 17	187 ± 65	12 ± 10^c^	15 ± 13^c^	138 ± 39^c^	7 ± 3^b^	10 ± 4^b,f^	56 ± 13^a^
Round 5	17 ± 10	26 ± 17	178 ± 58	12 ± 15	17 ± 15^c^	138 ± 53	5 ± 6^b^	10 ± 5^b,f^	58 ± 18^a^
Round 6	16 ± 10	28 ± 20	189 ± 58	9 ± 12	22 ± 12	141 ± 32	4 ± 4^b^	5 ± 4^a^	55 ± 16^a^

*TD* delay to desaturation, *FD* fast desaturation, *RT* round total; post hoc statistical analysis presented in the table

^a^*p* < 0.05 bag and pad compared to spar

^b^*p* < 0.05 bag compared to spar

^c^*p* < 0.05 bag compared to pad

^d^*p* < 0.05 compared to round 4 within the same training session

^e^compared to round 5 within the same training session

^f^*p* < 0.05 compared to round 6 within the same training session

^g^*p* < 0.05 compared to round 3 within the same training session

### Statistical analysis

All data are reported as means ± standard deviation. Main and interaction effects are reported in the text and post hoc pairwise analysis in the subsequent tables. Statistical analysis was carried out using Jamovi software (version 2.3.13). Quantile–quantile plots were run to determine normality of the individual components of the NIRS and punches thrown during different training. For all data, the points in the plots followed a linear diagonal line and were deemed to be normally distributed. A 3 × 2 × 6 repeated measures ANOVA was run on each component to determine main and interaction effects for NIRS components. A 3 × 6 repeated measures ANOVA was run for punches thrown. When there was a significant main effect or interaction effect, then a Fisher's least significant difference post hoc test was used to determine where differences occurred. Significance was accepted at *p* < 0.05. Effect size for main effect and interaction was calculated as adjusted partial eta squared (adjn_p_^2^; (Mordkoff 2019)) to remove bias, where 0.01–0.059 was a small effect, 0.06–0.137 was a moderate effect and ≥ 0.140 was a large effect (Wojdala et al. 2022).

## Results

### Punches thrown

There was a significant main difference and a large effect for total punches thrown during the time delay in muscle desaturation (*p* = 0.003, adjn_p_^2^ = 0.44; Table 1), fast desaturation (*p* < 0.001, adjn_p_^2^ = 0.72; Table 1) and total round (*p* < 0.001, adjn_p_^2^ = 0.84; Table 1) between different training.

### Skeletal muscle response during the round

#### Time delay to desaturation (TD)

There was a significant main difference and a large effect on TD by round (*p* = 0.016, adjn_p_^2^ = 0.21; Table 2) and by leg (*p* = 0.004, adjn_p_^2^ = 0.60; Table 2) but no difference between training for TD but a moderate effect (*p* = 0.249, adjn_p_^2^ = 0.09; Table 2).

**Table 2:** Skeletal muscle response during exercise

	TD (s)	HRS (BPM)	FDR (%.s^−1^)	FDReN (%Δ.p^−1^)	DD (s)	MAD FD	HRE (BPM)	MAD Eq
Round 1								
Lead leg—bag	11.8 ± 8.4^h^^,j,k^	125 ± 16 ^a,h,i,j,k,l^	– 1.71 ± 1.01^a,h,i,j^	– 1.49 ± 0.62^a^	23.3 ± 14.2^h^^,i^	7.1 ± 4.5^h^^,i,j,k,l^	152 ± 20^l^	2.0 ± 0.4^ l^
Rear leg—bag	9.0 ± 6.2	112 ± 23 ^h^^,i, j,k,l^	– 2.77 ± 1.94^ h^^,i,j,k,l^	– 2.54 ± 1.12^ h^^,i,j,k,l^	12.9 ± 5.6^i,j,k,l^	6.0 ± 4.2	131 ± 19^h^^,i,j,k,l^	2.6 ± 1.2
Lead leg—pad	11.9 ± 4.3^a,l^	124 ± 15^a,h,i,j,k,l^	– 1.59 ± 0.55^a,l^	– 1.93 ± 0.80^a^	24.8 ± 7.3^a^	7.4 ± 3.0^h^^,i,j,k,l^	148 ± 14^a,h,i,j,k,l^	3.1 ± 1.8
Rear leg—pad	6.8 ± 3.9	110 ± 20 ^h^^,i,j,k,l^	– 2.67 ± 1.62^ h^^,i,j,k,l^	– 3.36 ± 1.62^ h^^,i, j,l^	14.7 ± 5.3^h^^,i,k^	5.9 ± 2.8 ^i,j^	132 ± 22^h^^,i,j,k,l^	2.6 ± 0.9
Lead leg—spar	12.9 ± 4.7^i,j^	133 ± 14^a,h,i,j,k,l^	– 1.66 ± 0.64^a,k^	– 5.98 ± 4.83^b,c^	22.4 ± 6.8^ k^	5.6 ± 3.1^j,k,l^	146 ± 28^h^^,i,j,k,l^	2.1 ± 0.9^j,k,l^
Rear leg—spar	9.1 ± 3.6 ^i,j,l^	130 ± 18 ^e,f,h,i,j,k,l^	– 2.30 ± 1.45^i,j,k^	– 7.61 ± 4.73^e,f,k^	22.7 ± 14.2	7.3 ± 4.3^ k^	149 ± 23^h^^,i,j,k,l^	2.1 ± 1.0
Round 2								
Lead leg—bag	15.7 ± 7.5^a^	144 ± 11^j,k,l^	– 0.95 ± 0.53^a^	– 1.07 ± 0.78^a^	29.7 ± 11.9^a^	5.1 ± 3.0	159 ± 18	3.5 ± 3.1
Rear leg—bag	11.7 ± 5.7	141 ± 9^j,k,l^	– 2.25 ± 2.23^i,j,l^	– 2.43 ± 2.60^i,j,k,l^	20.7 ± 8.8	5.5 ± 4.4	151 ± 10^j,k,l^	2.2 ± 1.0
Lead leg—pad	11.2 ± 5.8^ l^	150 ± 10^j,k,l^	– 1.14 ± 0.38	– 1.49 ± 0.73	20.9 ± 7.2	3.2 ± 2.1	158 ± 12^k^^,l^	3.0 ± 1.0
Rear leg—pad	8.7 ± 4.1	152 ± 13^j,k,l^	– 1.23 ± 0.49	– 1.77 ± 0.85	25.4 ± 5.3^ l^	4.1 ± 2.7	159 ± 15^k^^,l^	3.5 ± 2.0
Lead leg—spar	15.1 ± 7.1 ^i,j^	156 ± 16^a,j,k,l^	– 1.54 ± 0.74^ k^	– 6.08 ± 4.45^b, c^	20.8 ± 6.7^ k^	5.3 ± 2.6^ k^^, l^	161 ± 21^i,j,k,l^	2.4 ± 0.6^k^^,l^
Rear leg—spar	11.9 ± 4.1 ^i,j,l^	153 ± 16^j,k,l^	– 1.61 ± 0.78	– 7.29 ± 4.41^e,f,k^	22.9 ± 5.7^ k^	4.7 ± 3.2	161 ± 20^k^^,l^	2.2 ± 0.4
Round 3								
Lead leg—bag	15.6 ± 10.2	149 ± 11^ l^	– 0.78 ± 0.45^a^	– 0.79 ± 0.39	35.2 ± 11.6^a,j,l^	4.6 ± 3.7	161 ± 16	2.5 ± 0.8^l^
Rear leg—bag	11.9 ± 5.1	148 ± 15^ l^	– 1.48 ± 0.76	– 1.32 ± 0.70	25.3 ± 3.9	6.6 ± 5.4	159 ± 10^l^	2.3 ± 0.7
Lead leg—pad	14.1 ± 10.4	156 ± 10^j,k,l^	– 1.28 ± 0.45	– 1.40 ± 0.72^k^	19.8 ± 8.8	3.3 ± 1.6	162 ± 10^k^^,l^	3.0 ± 2.1
Rear leg—pad	12.0 ± 11.9	154 ± 10^j^	– 1.17 ± 0.53	– 1.34 ± 0.73	25.5 ± 9.8	3.6 ± 1.7	163 ± 12	2.9 ± 1.5
Lead leg—spar	24.6 ± 11.1^k^	164 ± 25^j,k,l^	– 1.05 ± 0.58	– 4.07 ± 3.86	25.5 ± 8.3^b,d,k^	4.3 ± 3.4	168 ± 25	2.2 ± 0.7^k^^,l^
Rear leg—spar	19.6 ± 10.0	163 ± 26^k^^,l^	– 1.27 ± 1.18	– 5.82 ± 9.45	23.9 ± 13.6	4.1 ± 3.6	166 ± 27	2.7 ± 1.5
Round 4								
Lead leg—bag	16.2 ± 7.9	159 ± 15	– 0.98 ± 0.56	– 0.93 ± 0.62	25.4 ± 10.6	3.9 ± 3.3	167 ± 11	3.3 ± 2.3
Rear leg—bag	15.0 ± 10.2	152 ± 9	– 1.46 ± 0.78	– 1.63 ± 1.02	23.5 ± 12.0	6.6 ± 5.4	162 ± 9^g^^,l^	3.1 ± 2.1
Lead leg—pad	16.3 ± 11.0	163 ± 8	– 1.20 ± 0.61	– 1.74 ± 1.18^k^	19.8 ± 15.3	2.7 ± 2.5	169 ± 7	3.2 ± 0.9
Rear leg—pad	14.1 ± 9.4	163 ± 9	– 1.07 ± 0.61	– 1.62 ± 0.97	26.0 ± 18.4	3.2 ± 2.2	168 ± 8	3.1 ± 1.4
Lead leg—spar	22.2 ± 9.4	179 ± 17^a,b,c^	– 1.02 ± 0.48	– 3.47 ± 4.01	27.6 ± 8.0	3.5 ± 1.7	184 ± 14^b,c^	2.9 ± 0.9
Rear leg—spar	20.0 ± 10.9	176 ± 16^e,f^	– 1.20 ± 1.00	– 4.42 ± 6.13	23.9 ± 10.1	3.9 ± 4.1	182 ± 15^e,f^	2.6 ± 1.1
Round 5								
Lead leg—bag	17.2 ± 9.7^a,l^	155 ± 12	– 1.00 ± 0.48	– 1.06 ± 0.54	24.9 ± 10.4	3.5 ± 1.7	166 ± 11	3.4 ± 1.8^a^
Rear leg—bag	12.7 ± 7.0	154 ± 10	– 1.57 ± 1.14	– 1.60 ± 0.84	22.9 ± 9.0	4.7 ± 2.9	162 ± 10^l^	2.6 ± 1.2
Lead leg—pad	13.7 ± 6.1	163 ± 10	– 1.21 ± 0.68	– 2.98 ± 0.80^l^	21.9 ± 8.4	3.0 ± 1.8	168 ± 8	3.6 ± 1.8
Rear leg—pad	12.8 ± 5.3	162 ± 6	– 1.35 ± 1.06	– 2.39 ± 2.71	24.5 ± 13.0	4.5 ± 4.7	168 ± 7	2.9 ± 2.1
Lead leg—spar	15.6 ± 3.5	178 ± 14^b,c^	– 0.73 ± 0.58	– 2.67 ± 2.02	36.0 ± 13.3^c,e,l^	2.4 ± 1.8	181 ± 12^b,c^	4.1 ± 1.8^a^
Rear leg—spar	15.8 ± 9.0	178 ± 11^e,f^	– 0.75 ± 0.43^f,g,l^	– 2.55 ± 1.39	31.6 ± 8.8^l^	2.9 ± 2.7	181 ± 11^e,f^	2.8 ± 0.7
Round 6								
Lead leg—bag	13.1 ± 8.0	163 ± 11	– 1.08 ± 0.48	– 1.14 ± 0.61	24.0 ± 11.8	4.1 ± 2.3	172 ± 12	3.1 ± 0.7^a^
Rear leg—bag	13.2 ± 8.9	161 ± 10	– 1.42 ± 0.55	– 1.64 ± 0.95	21.8 ± 6.4	5.0 ± 2.9	168 ± 9	2.0 ± 0.7
Lead leg—pad	19.0 ± 28.5^a^	161 ± 9	– 0.87 ± 0.36	– 1.19 ± 0.67	26.1 ± 11.2	2.8 ± 1.8	170 ± 9	4.3 ± 2.0^a^
Rear leg—pad	22.9 ± 28.4	163 ± 9	– 1.37 ± 0.61	– 1.55 ± 0.68	19.9 ± 7.0	4.1 ± 2.5	167 ± 10	2.7 ± 1.6
Lead leg—spar	17.8 ± 8.3	178 ± 12^b,c^	– 1.01 ± 0.64^a^	– 5.05 ± 4.00^b,c^	23.5 ± 14.4	3.3 ± 2.2^a^	182 ± 13^b,c^	4.3 ± 2.2
Rear leg—spar	19.5 ± 9.2	179 ± 12^e,f^	– 1.61 ± 1.18	– 5.59 ± 3.62^e,f^	16.4 ± 7.9	5.2 ± 3.4	183 ± 15^e,f^	3.4 ± 2.2

*TD* time delay to fast desaturation, *HRS* heart rate at start of fast desaturation, *FDR* rate desaturation rate, *FDReN* fast desaturation rate normalised to duration and punches thrown [change in % saturation per punch], *DD* desaturation duration, *MAD FD* median absolute variation in SmO_2_ during fast desaturation, *HRE* heart rate at end of fast desaturation, *MAD Eq* median absolute variation in SmO_2_ during equilibrium). Post hoc statistical analysis presented in the table

Leg comparison:

^a^*p* < 0.05 rear leg compared to lead leg

Training comparison:

^b^*p* < 0.05 lead leg spar compared to lead leg bag

^c^lead leg spar compared to lead leg pad

^d^lead leg pad compared to lead leg bag

^e^*p* < 0.05 rear leg spar compared to rear leg bag

^f^rear leg spar compared to rear leg pad

^g^rear leg pad compared to rear leg bag

Round comparison:

^*h*^*p* < 0.05 compared to round 2 for same training modality and leg

^*i*^*p* < 0.05 compared to round 3 for same training modality and leg

^*j*^*p* < 0.05 compared to round 4 for same training modality and leg

^*k*^*p* < 0.05 compared to round 5 for same training modality and leg

^*l*^*p* < 0.05 compared to round 6 for same training modality and leg

#### Fast desaturation rate (FDR)

There was a significant main difference and a large effect on FDRe by round (*p* < 0.001, adjn_p_^2^ = 0.48; Table 2) and by leg (*p* = 0.003, adjn_p_^2^ = 0.60; Table 2) but no difference between training for FDRe and a trivial effect (*p* = 0.600, adjn_p_^2^ = 0.00; Table 2). When FDRe was normalised to duration of the fast desaturation and the number of punches thrown in the fast desaturation (FDReN), there was a significant main difference and a large effect on FDReN by round (*p* = 0.016, adjn_p_^2^ = 0.21; Table 2), training (*p* < 0.001, adjn_p_^2^ = 0.62; Table 2) and by leg (*p* = 0.003, adjn_p_^2^ = 0.62; Table 2).

#### Desaturation duration (DD)

There was a significant main difference and a large effect on DD by round (*p* = 0.019, adjn_p_^2^ = 0.19; Table 2) but not by leg (*p* = 0.080, adjn_p_^2^ = 0.25; Table 2) or training (*p* = 0.459, adjn_p_^2^ = 0.01; Table 2). There was a significant interaction for DD by round and training (*p* = 0.007, adjn_p_^2^ = 0.17; Table 2).

### Heart rate at skeletal muscle response during round

#### Fast desaturation start (FDS)

There was a significant main difference and a large effect on FDS by round (*p* < 0.001, adjn_p_^2^ = 0.86; Table 2), training (*p* < 0.001, adjn_p_^2^ = 0.74; Table 2) and by leg (*p* = 0.009, adjn_p_^2^ = 0.57; Table 2).

#### Fast desaturation end (FDE)

There was a significant main difference and a large effect on FDE by round (*p* < 0.001, adjn_p_^2^ = 0.74; Table 2), training (*p* = 0.018, adjn_p_^2^ = 0.34; Table 2) and by leg (*p* = 0.002, adjn_p_^2^ = 0.71; Table 2).

### Skeletal muscle response during recovery

Resaturation delay (RD): There was a significant main difference and a large effect on RD by leg (*p* = 0.045, adjn_p_^2^ = 0.34; Table 3) but no significant main difference and a trivial effect by round (*p* = 0.868, adjn_p_^2^ = 0.00; Table 3) and no significant main difference but a moderate effect by training (*p* = 0.095, adjn_p_^2^ = 0.17; Table 3).

**Table 3:** Skeletal muscle recovery between rounds

	RD (s)	HRSF (BPM)	FRR (%.s^−1^)	RDD (s)	MAD FR	HREF (BPM)	MAD SR
Round 1							
Lead leg—bag	12.7 ± 9.9	172 ± 17^h^^,i,j,k,l^	0.85 ± 0.36^h^^,i^	28.0 ± 16.0	4.2 ± 2.6	166 ± 12^j,k,l^	2.6 ± 1.5
Rear leg—bag	12.7 ± 10.2	168 ± 34	0.88 ± 0.49	38.0 ± 11.1	5.1 ± 3.2	171 ± 12	2.5 ± 3.1
Lead leg—pad	7.8 ± 7.1	176 ± 12^h^^,i,j,k^	0.88 ± 0.27	31.2 ± 9.6	4.5 ± 2.3	167 ± 11^i,k,l^	2.5 ± 1.3
Rear leg—pad	4.2 ± 3.4	178 ± 11^i,j,k^	1.19 ± 0.82	27.3 ± 12.2^g^	4.7 ± 2.5	172 ± 12^i,k,l^	3.2 ± 1.3
Lead leg—spar	10.4 ± 5.4	179 ± 9^h^^,i,j,k,l^	0.89 ± 0.39^l^	34.9 ± 10.2^f^	5.1 ± 3.2^ l^	167 ± 11^h^^,i,j,k,l^	2.9 ± 1.9
Rear leg—spar	8.9 ± 6.6	174 ± 12^h^^,i,j,k,l^	0.83 ± 0.33	37.0 ± 9.7	4.3 ± 2.0^ k^	176 ± 14^j,l^	3.2 ± 2.2
Round 2							
Lead leg—bag	11.3 ± 8.7	177 ± 14^d,i,j,l^	0.64 ± 0.24^a^	32.5 ± 10.4	4.0 ± 1.6^a,j,k,l^	173 ± 10^ l^	2.2 ± 1.2
Rear leg—bag	7.8 ± 6.6	177 ± 17^e,g,j,k,l^	0.88 ± 0.40	37.2 ± 12.2	6.2 ± 1.9	175 ± 9	3.2 ± 2.5
Lead leg—pad	8.0 ± 5.2	183 ± 11^j,k^	0.91 ± 0.15	27.3 ± 11.2	4.0 ± 1.8	174 ± 14	2.7 ± 1.3
Rear leg—pad	8.0 ± 9.0	184 ± 12^i,j^	0.87 ± 0.50	25.0 ± 11.8	4.4 ± 2.8	177 ± 10	3.9 ± 1.9
Lead leg—spar	10.6 ± 6.4	179 ± 23^b,c,j,k,l^	0.93 ± 0.39^j,k,l^	36.6 ± 12.7	4.8 ± 2.5^ k^^, l^	179 ± 12^b,c^	3.2 ± 1.2
Rear leg—spar	7.1 ± 4.7	178 ± 25^ l^	0.71 ± 0.26	38.4 ± 6.9	4.8 ± 1.9	185 ± 11^e,f^	2.9 ± 1.8
Round 3							
Lead leg—bag	14.7 ± 8.6	184 ± 14^b,d^	0.62 ± 0.18^a^	37.0 ± 11.4	3.7 ± 1.7	175 ± 11	2.5 ± 2.3
Rear leg—bag	11.3 ± 7.5	180 ± 18^e,k^	0.83 ± 0.31	34.4 ± 9.9	4.4 ± 3.4	179 ± 9	4.0 ± 2.6
Lead leg—pad	7.7 ± 6.1	189 ± 10	0.99 ± 0.36	29.2 ± 16.2	3.5 ± 1.8	176 ± 11	3.5 ± 1.6
Rear leg—pad	6.5 ± 7.9	188 ± 9	0.80 ± 0.23	31.8 ± 13.1	3.9 ± 2.1	182 ± 10	3.7 ± 2.0
Lead leg—spar	9.5 ± 9.1	191 ± 8	0.81 ± 0.41	35.2 ± 13.1	4.3 ± 3.7	182 ± 10	3.0 ± 2.5
Rear leg—spar	8.3 ± 6.3	190 ± 9	0.77 ± 0.36	38.2 ± 6.7	3.8 ± 2.5	186 ± 10^e^	3.0 ± 2.2
Round 4							
Lead leg—bag	12.2 ± 10.5	183 ± 13^d^	0.66 ± 0.19	37.4 ± 15.1	3.7 ± 2.3	178 ± 7	3.0 ± 1.8
Rear leg—bag	10.5 ± 6.9	183 ± 17^e,l^	0.79 ± 0.26	32.7 ± 10.8	4.7 ± 2.7	179 ± 9	2.8 ± 1.5
Lead leg—pad	9.0 ± 7.5	188 ± 12	0.73 ± 0.23	31.6 ± 10.7	2.7 ± 1.4	178 ± 10	3.3 ± 1.8
Rear leg—pad	9.0 ± 6.9	189 ± 11	0.80 ± 0.30	30.5 ± 7.3	4.0 ± 2.7	179 ± 12	4.3 ± 2.4
Lead leg—spar	9.7 ± 7.0	194 ± 9^b,c,l^	0.71 ± 0.30	36.5 ± 11.4	3.2 ± 2.0	185 ± 8^a,b,c^	2.5 ± 1.8
Rear leg—spar	10.5 ± 9.3	193 ± 12	0.79 ± 0.34	35.0 ± 10.6	3.1 ± 2.1	189 ± 11^e^	3.3 ± 1.9
Round 5							
Lead leg—bag	11.9 ± 8.9	185 ± 16^d^	0.70 ± 0.26^a^	30.5 ± 12.1	4.2 ± 2.5	179 ± 11	2.7 ± 1.5
Rear leg—bag	13.1 ± 9.4	187 ± 15	0.85 ± 0.25	25.4 ± 11.8	4.1 ± 2.4	181 ± 9	2.5 ± 1.8
Lead leg—pad	8.1 ± 6.5	189 ± 11	0.72 ± 0.32	31.7 ± 10.9	3.3 ± 1.4	182 ± 15	2.6 ± 1.0
Rear leg—pad	3.7 ± 4.3	189 ± 9	0.84 ± 0.24	28.2 ± 16.6	3.2 ± 1.5	180 ± 9	3.4 ± 1.7
Lead leg—spar	8.7 ± 4.9	191 ± 13^b,c^	0.68 ± 0.26^a^	41.3 ± 5.0^b,c^	3.1 ± 2.1	184 ± 10^b^	3.7 ± 2.1
Rear leg—spar	10.6 ± 10.0	191 ± 12	0.85 ± 0.25	35.8 ± 13.6	3.2 ± 2.0	185 ± 10^e^	3.4 ± 1.6
Round 6							
Lead leg—bag	13.5 ± 10.4	188 ± 15	0.62 ± 0.25	27.1 ± 9.3	3.6 ± 2.7	182 ± 10	2.0 ± 1.0
Rear leg—bag	11.6 ± 7.5	188 ± 15	0.93 ± 0.45	28.3 ± 12.6	3.9 ± 2.3	185 ± 10	2.5 ± 1.6
Lead leg—pad	12.8 ± 10.9	189 ± 12	0.85 ± 0.28	23.8 ± 16.1	2.7 ± 1.6^a^	182 ± 15	3.4 ± 2.4
Rear leg—pad	9.7 ± 7.0	189 ± 11	0.88 ± 0.26	34.4 ± 9.2	4.8 ± 2.7	184 ± 9	3.5 ± 1.6
Lead leg—spar	4.1 ± 5.5	191 ± 14^c^	0.69 ± 0.28	38.2 ± 6.7^b^	3.0 ± 1.8	182 ± 18^b^	4.2 ± 2.0
Rear leg—spar	10.4 ± 6.3	191 ± 12^e,f^	0.72 ± 0.27	37.5 ± 7.3	3.3 ± 2.3^f^	184 ± 14^f^	3.6 ± 1.6

*RD* resaturation delay, *HRSF* heart rate at start of fast resaturation, *FRR* fast resaturation rate, *FRD* fast resaturation duration, *MAD FR* median absolute variation in SmO_2_ during fast resaturation, *HREF* heart rate at end of fast resaturation, *MAD SR* median absolute variation in SmO_2_ during slow resaturation). Post hoc statistical analysis presented in the table

Leg comparison:

^a^*p* < 0.05 rear leg compared to lead leg

Training comparison

^*b*^*p* < 0.05 lead leg spar compared to lead leg bag

^c^lead leg spar compared to lead leg pad

^d^lead leg pad compared to lead leg bag

^e^*p* < 0.05 rear leg spar compared to rear leg bag

^f^rear leg spar compared to rear leg pad

^g^rear leg pad compared to rear leg bag

Round comparison

^*h*^*p* < 0.05 compared to round 2 for same training modality and leg

^*i*^*p* < 0.05 compared to round 3 for same training modality and leg

^*j*^*p* < 0.05 compared to round 4 for same training modality and leg

^*k*^*p* < 0.05 compared to round 5 for same training modality and leg

^*l*^*p* < 0.05 compared to round 6 for same training modality and leg

#### Fast resaturation rate (FRR)

There was a significant main difference and a large effect on FRR by rounds (*p* = 0.003, adjn_p_^2^ = 0.30; Table 3) but no significant main difference and a large effect by leg (*p* = 0.054, adjn_p_^2^ = 0.34; Table 3) and no significant main difference but a moderate effect by training (*p* = 0.175, adjn_p_^2^ = 0.14; Table 3).

#### Fast resaturation duration (FRD)

There was a significant main difference and a large effect on FRD by training (*p* = 0.002, adjn_p_^2^ = 0.45; Table 3) but no significant main difference and a trivial effect by leg (*p* = 0.853, adjn_p_^2^ = 0.00; Table 3) and by rounds (*p* = 0.778, adjn_p_^2^ = 0.00; Table 3).

### Heart rate at skeletal response during recovery

#### Heart rate at start of fast resaturation (HRSF)

There was a significant main difference and a large effect on HRSF by training (*p* = 0.015, adjn_p_^2^ = 0.46; Table 3) and by round (*p* < 0.001, adjn_p_^2^ = 0.68; Table 3) but no significant main difference and a moderate effect by leg (*p* = 0.381, adjn_p_^2^ = 0.07; Table 3).

Heart rate at end of fast resaturation (HREF): There was a significant main difference and a large effect on HREF by round (*p* < 0.001, adjn_p_^2^ = 0.65; Table 3), training (*p* = 0.006, adjn_p_^2^ = 0.55; Table 3) and leg (*p* = 0.002, adjn_p_^2^ = 0.80; Table 3).

### Median absolute deviation (MAD)

#### During exercise

There was a significant main difference and a large effect on MAD during fast desaturation by round, with variation across fast desaturation becoming smaller across rounds (*p* < 0.001, adjn_p_^2^ = 0.55; Table 3) and by leg (*p* = 0.024, adjn_p_^2^ = 0.41; Table 3) but no main difference between the training during fast desaturation with a moderate effect (*p* = 0.249, adjn_p_^2^ = 0.15; Table 3). During the steady state, there was a significant main difference and a large effect on MAD by leg (*p* = 0.004, adjn_p_^2^ = 0.60; Table 3), with a significant leg by round interaction (*p* = 0.009, adjn_p_^2^ = 0.23; Table 3) but no significant main effect across rounds (*p* = 0.086, adjn_p_^2^ = 0.13; Table 3) or between the different training (*p* = 0.366, adjn_p_^2^ = 0.02; Table 3).

#### During recovery

There was a significant main difference and a large effect on MAD during fast resaturation by round, with variation across fast resaturation becoming smaller across rounds (*p* < 0.001, adjn_p_^2^ = 0.22; Table 3) but not by leg (*p* = 0.094, adjn_p_^2^ = 0.23; Table 3) or training (*p* = 0.417, adjn_p_^2^ = 0.01; Table 3). During the slow resaturation, there was no significant main difference on MAD by leg (*p* = 0.191, adjn_p_^2^ = 0.12; Table 3), across rounds (*p* = 0.700, adjn_p_^2^ = 0.00; Table 3) or between the different training (*p* = 0.117, adjn_p_^2^ = 0.15; Table 3).

## Discussion

The aim of this study was to look at the difference in the rectus femoris in three different boxing training sessions in professional boxers. There is a similar response in the rectus femoris muscle oxygenation across the three training methods, but the punch count is much lower in sparring. Across a round, the rectus femoris muscle response can be modelled as initial delay, fast desaturation and new equilibrium, suggesting a continuous activity for this muscle rather than being intermittent. Across sparring, there is an increased variability during equilibrium which is not present in bag or pad. Recovery from a round can also be modelled as initial delay, fast resaturation and slow resaturation. The rate of fast resaturation is reduced in later rounds reflecting poor muscular recovery in professional boxers. There is a need to ensure that athlete conditioning improves oxygenation dynamics in the rectus femoris muscle to prevent model deterioration as a competition continues.

For each training session, the time delay for muscle desaturation was similar (Table 2, Fig. 1). The time delay reflects where the muscular work being done is not increasing mitochondrial activity beyond the capacity of oxygen availability (myoglobin reserve and oxygenated haemoglobin in the microcirculation). Across rounds, the trend is for the time delay to be extended with all training (Table 2), which would suggest impaired mitochondrial activation in the skeletal muscle beyond the oxygen availability. Heart rate at the start of fast desaturation/end of time delay is elevated (Table 2) across later rounds with the different training, suggesting higher systemic blood flow. However, this might not reflect microcirculatory flow at the muscle (Ferreira et al. 2005). From round 4 on, there is a significantly greater heart rate at the start of fast desaturation/end of time delay during sparing compared to either bag or pad training (Table 2). In amateur boxing, average heart rate across a round has been shown to increase by 8 bpm in round 2 and 11–14 bpm in round 3 during simulated or sparring (De Lira et al. 2013; Finlay et al. 2018, 2020; Lal Khanna and Manna 2006), which is lower than the rise seen across the time delay in the first three rounds. The increased heart rate across the time delay and increased duration of time delay reflect the inability of the rectus femoris muscle to recover from the physiological stress of the previous round. Potentially this could lead to an accumulated fatigue.

For each training modality, the rate of fast desaturation in the muscle was similar (Table 2, Fig. 1). This represents where oxygen use within the mitochondria outstrips the delivery to the skeletal muscle, leading to utilisation of the skeletal muscle myoglobin reserve. During maximal exercise, myoglobin contributes approx. 50% to the total NIRS muscle desaturation signal (M. L. Davis and Barstow 2013). The fall in desaturation of myoglobin has been shown to start after 15 s in dog muscle, similar to time delay reported here, and is suggested to facilitate oxygen flux and intracellular oxygen diffusion (Gayeski et al. 1985). There is a decrease in rate of fast desaturation across rounds which may reflect poor recovery between rounds in professional boxers (Dupont et al. 2004). Whilst the rate of desaturation is changing across the rounds, the duration of the fast desaturation period is remaining constant (Table 2, Fig. 1). The variation in the SmO_2_ data across the fast desaturation gets less as the rounds continue (Table 3), which may highlight impaired mitochondrial activation or reduced intensity within the round even though punch count remains similar (Table 1). When the fast desaturation is normalised to punches thrown during that time frame, we see a significantly different response in the rate of deoxygenation per punch compared to bags or pads (Table 2). This may reflect that there is a lot more lower body movement demand in sparring to deliver a punch compared to bag or pad sessions.

The equilibrium component represents where skeletal muscle oxygen utilisation and delivery are balanced but at a lower level of overall muscle oxygenation than pre exercise (Fig. 1). The variation across the steady state represents efficiency during the exercise, with less variation reflecting greater efficiency. Variation is small in the muscle across all training in round 1 (Table 3, Fig. 1) and was largely unaffected during bag and pad sessions (Table 3, Fig. 1). In sparring, the later rounds became more variable compared to the earlier rounds (Table, Fig. 1). In amateur boxing, an increase in player load in round 2 and 3 has been associated with fatigue and loss of efficiency (Finlay et al. 2020), which may reflect the loss of equilibrium. This suggests that there is a greater difficulty in achieving an equilibrium in the later rounds of sparring which will lead to less contraction for movement or force production from the rectus femoris muscle. Boxing is normally considered an intermittent sport (Finlay et al. 2018) but the establishment of a stable equilibrium in the rectus femoris muscle across rounds with all training suggests that the mitochondria within this muscle are constantly metabolically active like fixed work rate exercise (Hong et al. 2022).

The time delay before reoxygenation is very similar with training and rounds (Table 3, Fig. 1). This reflects a balance between the oxygen flow and mitochondrial activity within the muscle. Skeletal muscle recovery then moves into a fast linear stage where most of the reoxygenation occurs. There is a significant decrease in the rate of fast resaturation across rounds although the duration remains similar (Table 3, Fig. 1). This reflects mitochondrial activity decreasing whilst flow to the skeletal muscle is still high, allowing reoxygenation to occur. Given the decrease in fast resaturation rate across rounds, skeletal muscle recovery for athletes is being impeded which impacts rectus femoris desaturation in subsequent rounds (Table 2), but not on punches thrown (Table 1). The greater heart rate seen in sparring compared to other training modalities is seen in the ability to recover. The heart rate at the start and end of fast resaturation is higher across later rounds and is different in sparring compared to the other training modalities (Table 3). In simulated amateur boxing, heart rate is greater after 60 s recovery in round 2 and 3 compared to round 1 (De Lira et al. 2013), similar to the resaturation period for bags and pads but smaller than we see in sparring. Given that this covers the first 40–50 s of recovery which suggests that professional boxers are not utilising the 60 s breaks between rounds to allow the rectus femoris muscle to effectively recover.

## Limitations

This is an observational study of the effects of different training on the rectus femoris muscle. As such no attempt was made to control the athletes training load before taking part in the study. This may influence heart rate recovery across the rounds (Borresen and Lambert 2007). Pad work was carried out by the same coach, which means it may not be fully representative of typical pad work in boxing. The sessions may have had a higher or lower intensity than the athletes were accustomed to. The overall punches thrown were significantly less in sparring compared to bag and pad sessions (Table 1). This may suggest a different physiological load in bag and pad work compared to sparring if you consider punches thrown to be the only work carried out during a session. However, the lower body muscles are recruited for movement as well as for force development. To date, the current study gives the most accurate representation of the three training types as they are used by professional boxers and coaches as we have not sought to control total number or type of punches used.

## Conclusion and practical implications

Given that punch force kinetic chain starts from the ground and through the rectus femoris (Lenetsky et al. 2020), it is easy to view punches thrown as a marker of the total workload. However, we see a significantly lower number of punches thrown in sparring (Table 1), yet the oxygenation response of the rectus femoris muscle is similar between training (Table 2). Further, the rectus femoris muscle is constantly active during the 3 min round, suggesting continuous activation rather than intermittent use. The pattern of desaturation, although not analysed in this way, is seen in studies that have used the continuous 3-min critical power cycle test or 1500 m cycle time trials (A. Feldmann and Erlacher 2021; Hesford et al. 2013) Given that round duration is similar to elite 1500 m run times, which is viewed as a predominantly endurance event, there is a need to reconsider conditioning for professional boxers. We present a new model of boxing physiology which shows three stages in muscle oxygen use, desaturation delay, fast desaturation and equilibrium (Fig. 1, Table 2), with a similar three component model for recovery (Fig. 1, Table 3). More research is needed to understand how these components change with structured training. However, given that the steady state exercise is proposed to be energetically efficient (Ferretti et al. 2017), an earlier onset of equilibrium with less variation would be a desirable.

## Data Availability

Data will be available upon reasonable request.
